# Resequencing of genes for transforming growth factor β1 (*TGFB1*) type 1 and 2 receptors (*TGFBR1*, *TGFBR2*), and association analysis of variants with diabetic nephropathy

**DOI:** 10.1186/1471-2350-8-5

**Published:** 2007-02-23

**Authors:** Amy Jayne McKnight, David A Savage, Chris C Patterson, Denise Sadlier, A Peter Maxwell

**Affiliations:** 1 Nephrology Research Group, Queen's University of Belfast, Belfast, UK; 2 Epidemiology Research Groups, Queen's University of Belfast, Belfast, UK; 3 Faculty of Life Sciences, University of Manchester, Manchester, UK; 4 Conway Institute of Biomolecular and Biomedical Research, University College Dublin, Dublin, Ireland

## Abstract

**Background:**

Diabetic nephropathy is the leading cause of end stage renal failure in the western world. There is substantial epidemiological evidence supporting a genetic predisposition to diabetic nephropathy, however the exact molecular mechanisms remain unknown. Transforming growth factor (TGFβ1) is a crucial mediator in the pathogenesis of diabetic nephropathy.

**Methods:**

We investigated the role of five known single nucleotide polymorphisms (SNPs) in the *TGFB1 *gene for their association with diabetic nephropathy in an Irish, type 1 diabetic case (n = 272) control (n = 367) collection. The activity of TGFβ1 is facilitated by the action of type 1 and type 2 receptors, with both receptor genes (*TGFBR1 *and *TGFBR2*) shown to be upregulated in diabetic kidney disease. We therefore screened *TGFBR1 *and *TGFBR2 *genes for genomic variants using WAVE™ (dHPLC) technology and confirmed variants by direct capillary sequencing. Allele frequencies were determined in forty-eight healthy individuals. Data for all SNPs was assessed for Hardy Weinberg equilibrium, with genotypes and allele frequencies compared using the χ^2 ^test for contingency tables. Patterns of linkage disequilibrium were established and common haplotypes estimated.

**Results:**

Fifteen variants were identified in these genes, seven of which are novel, and putatively functional SNPs were subsequently genotyped using TaqMan™, Invader™ or Pyrosequencing^® ^technology. No significant differences (*p *> 0.1) were found in genotype or allele distributions between cases and controls for any of the SNPs assessed.

**Conclusion:**

Our results suggest common variants in *TGFB1*, *TGFBR1 *and *TGFBR2 *genes do not strongly influence genetic susceptibility to diabetic nephropathy in an Irish Caucasian population.

## Background

Diabetic nephropathy is a major clinical complication of diabetes mellitus. Epidemiological evidence supporting a genetic contribution to this disease includes ethnicity differences [1], familial clustering [2,3] and the fact that only a subset of individuals with type 1 diabetes develops diabetic nephropathy regardless of metabolic control [4]. In addition, simulation studies have shown that environmental effects are insufficient to account for the familial aggregation of this disease [3,5].

Transforming growth factor beta (TGFβ1) is a multifunctional cytokine implicated in the pathogenesis of many forms of progressive renal disease, including diabetic nephropathy, by promoting renal hypertrophy and the accumulation of extracellular matrix [6]. Protein and mRNA levels of TGFβ1 are significantly increased in the renal glomeruli and tubulointerstitium of animal models of diabetes and in humans with diabetes [7]. TGFβ1 is transcriptionally activated by high extracellular glucose in murine glomerular mesangial cells [8]. Transgenic mice over-expressing TGFβ1 develop progressive renal failure, suggesting that chronically elevated levels of circulating TGFβ1 are integral to the pathogenesis of kidney disease [9,10]. Direct blockade of TGFβ protein by chronic administration of anti-TGFβ1 antibodies has been shown to decrease renal insufficiency [11]. In addition, antisense TGFβ1 oligonucleotides reduce the cellular hypertrophy and stimulation of matrix synthesis normally seen in renal cells exposed to high extracellular glucose [12].

The activity of TGFβ1 in regulating cell proliferation, differentiation and extracellular matrix production are mediated by a heterodimeric complex of type 1 and type 2 receptors. Upregulation of TGFβ1 receptors have been reported in animal models of glomerulosclerosis [13,14]. It has been proposed that upregulation of TGFβR2 induced by high extracellular glucose may contribute to distal tubular hypertrophy in diabetic nephropathy [15]. Isono and colleagues demonstrated that increased expression of TGFβR2 in the diabetic kidney is primarily due to stimulation of gene transcription rather than increased mRNA stability [16].

TGFβ1 is encoded by the *TGFB1 *gene located at chromosome 19q13.1 [17]. We have investigated the role of five known single nucleotide polymorphisms, which may influence *TGFB1 *gene expression (*TGFB1*: -800G>A, -509C>T, +72InsC, +869T>C, +915G>C) for their association with diabetic nephropathy. TGFβ receptors type 1 and type 2 are encoded by *TGFBR1 *and *TGFBR2 *genes respectively. At present there are over six hundred variants recorded in dbSNP for these genes, with little information available on the role of these variants in relation to renal complications of diabetes. We have screened the genomic draft sequence for the *TGFBR1 *and *TGFBR2 *genes in an Irish population to identify genomic variants. Allele frequencies were subsequently determined in a healthy control population and selected SNPs genotyped in a case-control collection. In summary, we investigated if putatively functional variants in three genes, *TGFB1, TGFBR1 and TGFBR2*, contribute to genetic susceptibility to diabetic nephropathy in type 1 diabetes.

## Methods

### Subjects

Ethical approval was obtained from the appropriate Research Ethics Committees in each country and written, informed consent obtained from individuals prior to conducting this study. The case and control groups used for this study (Table 1) have been described previously [18]. All patients were at least third generation Irish Caucasians diagnosed with type 1 diabetes mellitus before 31 years of age, and required insulin from diagnosis. Patients with nephropathy (cases, n = 272) had diabetes for at least 10 years before the onset of proteinuria (>0.5 g/24 h). Patients without nephropathy (controls, n = 367) had diabetes for at least 15 years, were not in receipt of antihypertensive medication, and had no evidence of non-diabetic renal disease. Patients with microalbuminuria were excluded from both groups.

**Table 1:** Clinical characteristics of cases (n = 272) and controls (n = 367) (Data are n, mean ± SD)

**Criteria**	**Cases**	**Controls**
**N**	272	367
**Gender**		
Male	61.4 %	40.3 %
Female	38.6 %	59.7 %
**Age at Diagnosis **(years)	17.1 ± 8.2	16.8 ± 8.1
*Duration of Diabetes (years)	26.9 ± 8.3	27.7 ± 9.0
**HbA1**_**C **_(%)	8.5 ± 1.7	8.4 ± 1.6
**BMI **(kg/m^2^)	26.0 ± 3.8	26.2 ± 3.5
**Systolic Blood Pressure **(mm Hg)	150.1 ± 22.6	126.9 ± 16.6
**Diastolic Blood Pressure **(mm Hg)	86.5 ± 11.4	76.1 ± 7.3

* Duration of diabetes was calculated from the date of diagnosis of diabetes to date of recruitment

* BMI = body mass index

### *In silico *analysis

For *TGFBR1*, the nucleotide sequence of draft clone RP11-96L7 for human chromosome 9 was downloaded from the National Centre for Biotechnology Information [19]. Similarly, the sequence for *TGFBR2 *was obtained for draft clone RP11-1024P17 on human chromosome 3. Reference mRNA (NM_004612; NM_003242) and protein (NP_004603; NP_003233) sequences were also downloaded from NCBI for *TGFBR1 *and *TGFBR2 *respectively. These were used to determine intron-exon boundaries for genomic DNA using Vector NTI Advance (suite 2, version 8, Informax Inc (Europe), Oxford, UK). The nomenclature for all identified variants follows the Human Genome Variation Society recommendations for coding sequences, updated 21^st ^May 2005 [20]. In addition, we have provided rs numbers for all previously identified SNPs and ss numbers for novel SNPs to facilitate ease of comparison between research groups.

### Amplification and mutation screening

6464 bases of *TGFBR1 *and 5204 bases of *TGFBR2 *genomic sequences were divided into fragments with an average size of approximately 500 base pairs, for PCR and screening purposes in 15 case and 15 control individuals. As the *TGFBR1 *and *TGFBR2 *gene sequences cover approximately 45 kb and 84 kb respectively from start to stop codon, only the coding regions of these genes (including all exons, exon-intron boundaries and untranslated regions) were screened to prioritise the identification of potentially functional gene variants. Each PCR product was then evaluated using WaveMaker v3.4 software (Transgenomic Ltd, Crewe, UK) and analysed on the WAVE™ (dHPLC) DNA Fragment Analysis System (Transgenomic Ltd) following the manufacturer's recommendations. Differentially separating fragments (representing DNA variants) were bidirectionally sequenced to identify variants using an ABI PRISM^® ^3100 Genetic Analyser (Applied Biosystems, Warrington, UK). Forty-eight healthy controls (n = 96 chromosomes) from the Young Hearts collection [21] (a healthy Irish Caucasian population) were genotyped by direct capillary sequencing (Applied Biosystems) to establish allele frequencies for all gene variants.

### Genotyping

Five SNPs were selected for genotyping in the TGFB1 gene as they have been previously suggested to influence the expression of TGFβ1, in addition to demonstrating a minor allele frequency greater than 5%. TaqMan assays were successfully designed for *TGFB1: *-800G>A (rs1800468), *TGFB1: *-509C>T (rs1800469), *TGFB1: *+869T>C (rs1982073) and *TGFB1: *+915G>C (rs1800471) SNPs, but proved problematic for *TGFB1: *+72InsC (rs1800999) due to the presence of a long C homopolymer. *TGFB1: *+72InsC was successfully genotyped using a biplex Invader™ assay (Third WAVE Technologies Inc, Madison, MI, USA). Genotyping was performed for receptor variants using Pyrosequencing^® ^technology according to the manufacturer's instructions (Biotage, Uppsala, Sweden). Details of the primer sequences used for resequencing purposes, together with the WAVE conditions and the oligos used for the genotyping assays are listed (Tables 2, 3, 4, 5) with further details readily available from the authors on request. 272 case and 367 control samples were available for genotyping *TGFB1 *SNPs, however fewer samples were available (241 cases and 322 controls) for genotyping *TGFBR2 *gene variants. Genotype frequencies were assessed for Hardy-Weinberg equilibrium using a χ^2 ^goodness-of-fit test. The χ^2 ^test for contingency tables was used to compare genotype and allele frequencies between case and control subjects with the level of significance set to p < 0.05. Haploview [22] was used to visualise linkage disequilibrium (LD) and haplotype blocks within each gene.

**Table 2:** Primer sequences used for screening *TGFBR1 *and *TGFBR2 *genes

***Primer Set***	Primer 1	Primer 2
***TGFBR1 *i**	tcctccttaaaaggttctgc	agaaagtcctcagatcccag
***TGFBR1 *ii**	ggaggctatttgggggtgt	gcgagcgccggtttctg
***TGFBR1 *iii**	actcacacagacacaccca	aagagcaggagcgagccag
***TGFBR1 *iv**	ctaagagcaacaaacagtgc	gtcacttcttgcctctaaacg
***TGFBR1 *v**	tgcaggaattgtgtaggattg	tggagctgacttattgattcg
***TGFBR1 *vi**	ctccccagtgagataaattc	aatcttgaagaagttcctag
***TGFBR1 *vii**	gcttactctgaggaactaaag	agatgcggttttgtcatgttg
***TGFBR1 *viii**	aagtattgtaggtcatgtgg	gatattttctggaagggcaac
***TGFBR1 *ix**	gtctgaaaggaggttcatc	caggaagagaatacactagg
***TGFBR1 *x**	gtgatcttttaatgccttgg	aacattggtttgactgcta
***TGFBR1 *xi**	caccagtaccctattgatgg	aaggagagttcaggcaaagc
***TGFBR1 *xii**	gcaactcagtcaacaggaag	gaatcaaggaaactctagtgg
***TGFBR1 *xiii**	agaaagtgatttactcct g	attcaaacatgaccatgc
***TGFBR1 *xiv**	ctttctcctaccaaaatgtgc	ctgaattaaaagctgccttcc
***TGFBR2 *i**	cctcctggctggcgagcg	ggaccaaacgtgccccgc
***TGFBR2 *ii**	aagcaaatggctactcaacc	acacatacatgcagagaacacc
***TGFBR2 *iii**	tgcgaatgctggagaacagg	ggaggacaccacctaacgtatg
***TGFBR2 *iv**	agctgaagtttgaaggaagagc	gcacacggttgttgtagttggt
***TGFBR2 *v**	catcatcttctactgctaccgc	ggttcccgttggatgtcctcat
***TGFBR2 *vi**	ggagttggggaaacaatactgg	gggtcaagtcgtgtaaaaaagg
***TGFBR2 *vii**	ctatctgtacctttctgtgc	ccaatacgatttgtcggatc
***TGFBR2 *viii**	gttacttagtgcttcatgctcc	ccttccagggtaacacaagata
***TGFBR2 *ix**	gtgttgggagtgttagtgtacc	ccgtaggtctaccacacactct
***TGFBR2 *x**	accaactcatggtgccctttgg	cggtatggaacttttctctg
***TGFBR2 *xi**	gctgtgttagcacttcctcagg	ggtttagaccccccgatcaaat
***TGFBR2 *xii**	tgtttgaggaccagtgttcccg	ccgaggactaacgagttcgtgt

**Table 3:** WAVE conditions used for screening *TGFBR1 *and *TGFBR2 *genes.

**Primer Set**	**Tm°C**	**Melt Range°C**	**Run Temperature°C**	**WAVE Buffer B (%)**
***TGFBR1 *i**	59.4	54 – 72	57	69–79
			59	67–77
			64	62–64
***TGFBR1 *ii**	63.3	60 – 72	63	68–78
			69	62–72
***TGFBR1 *iii**	69.2	58 – 75	69	68–78
			72	65–75
***TGFBR1 *iv**	55.9	54 – 65	55	68–78
			59	64–74
***TGFBR1 *v**	57.2	54 – 65	55	62–72
			59	58–68
			61	56–58
***TGFBR1 *vi**	56.1	54 – 65	56	68–78
			59	65–75
***TGFBR1 *vii**	56.5	54 – 68	55	69–79
			58	66–76
***TGFBR1 *viii**	57.0	54 – 65	57	67–77
***TGFBR1 *ix**	57.4	55 – 67	56	67–77
			58	64–75
			62	61–71
***TGFBR1 *x**	54.8	51 – 66	54	65–75
			57	62–72
***TGFBR1 *xi**	54.6	54 – 67	54	65–75
			58	60–70
***TGFBR1 *xii**	56.9	55 – 65	56	64–77
			58	62–72
***TGFBR1 *xiii**	55.8	52 – 62	56	67–77
***TGFBR1 *xiv**	55.2	54 – 63	55	67–77
			59	63–73
***TGFBR2 *i**	66.1	59–74	60	63–73
			65	58–68
			67	56–66
			70	53–63
***TGFBR2 *ii**	66.3	61 – 72	64	58–68
			66	56–66
			69	52–62
***TGFBR2 *iii**	58.5	57 – 61	59	61–71
			61	59–69
***TGFBR2 *iv**	59.8	55 – 65	56	67–77
			61	60–70
			63	58–68
***TGFBR2 *v**	60.5	57–64	62	58–68
***TGFBR2 *vi**	63.5	62–65	64	55–65
***TGFBR2 *vii**	59.7	53–71	59	57–67
			61	52–62
***TGFBR2 *viii**	59.7	53 – 71	59	66–76
			61	64–74
***TGFBR2 *ix**	61.0	60–61	61	57–67
***TGFBR2 *x**	57.0	51–66	52	63–73
			58	57–67
			62	53–63
			64	50–60
***TGFBR2 *xi**	54.4	53 – 66	54	67–77
			56	65–75
			60	61–71
***TGFBR2 *xii**	57.0	56–60	59	63–73

**Table 4:** TaqMan primers, probes, quencher and annealing temperature for relevant assays.

**Primer set**	**Primer 1**	**Primer 2**	**FAM™ Labelled Probe**	**Vic™ Labelled Probe**	**Fluorescent Quencher**	**Anneal **(1 minute)
*TGFB1 *-800G>A	gctatcgcctgcacacagc	aggacagaagcggtcccat	tgcctccaacgtcaccaccatc	tctgcctccaacatcaccaccatc	TAMRA	62°C
*TGFB1 *-509C>T	ttagccacatgggaggtgct	ccaggcggagaaggcttaa	acccttccatccctcaggtgtcct	ccctcccatccttcaggtgtcctg	TAMRA	62°C
*TGFB1 *+869T>C	caccacaccagccctgttc	ccaggcgtcagcaccagta	agcagcggcagca	cagcagcagcagc	None	60°C
*TGFB1 *+915G>C	Developed by Applied Biosystems (USA) as a Research and Development Kit				None	56°C

*TGFBR1*+72InsC was genotyped by a commercial Invader kit designed and manufactured by Third WAVE technologies (Madison, MI, USA)

**Table 5:** Pyrosequencing primers, dispensation order and sequence to analyse for relevant assays

**Primer Set**	**Forward Primer**	**Reverse Primer**	**Sequence Primer**	**Dispensation order**	**Sequence to Analyse**
TGFBR2 c.*747C>G	tcctgtgtgcccttatttctc	tgaaggtaaaaagtggggttc	agtttctaaactaggttgag	tcgagagtctac	c/ggagagtttctaaac
TGFBR2 c.1149G>A	gatcacactccatgtggg	ccagacgcagggaaagc	agagctccaatatcctc	tgatgagacgac tac	g/atgaagaacgacctaacc

## Results

We have submitted our annotated sequencing data for *TGFBR1 *and *TGFBR2 *genes as GenBank accession numbers DQ383416 – DQ383424 and DQ377553 – DQ377559 respectively. A total of fifteen variants were identified in these genes (*TGFBR1*, n = 5; *TGFBR2*, n = 10) of which eight were previously recorded in dbSNP; we have obtained unique NCBI identifiers for all novel SNPs (n = 7; Table 6).

**Table 6:** Minor allele frequencies of identified variants in *TGFBR1 *and *TGFBR2 *genes, based on genotyping of 48 healthy control individuals. Accepted GenBank accession numbers for the reference sequences describing *TGFBR1 *and *TGFBR2 *gene variants are DQ383416 – DQ383424 and DQ377553 – DQ37759 respectively.

**Variant**	**Unique NCBI Identifier**	**Minor Allele Frequency (%)**
^*a*^*TGFBR1*: c.694A>C	ss50394789	4.3
^*a*^*TGFBR1*: c.*899T>C	ss50394790	2.2
^*a*^*TGFBR1*: c.*921T>G	ss50394793	4.3
^*a*^*TGFBR1*: c.*978G>A	ss50394791	1.1
^*a*^*TGFBR1*: c.*1004A>T	ss50394792	2.2
*TGFBR2*: c.263+7A>G	rs1155705	10.8
^*a*^*TGFBR2*: c.263+17A>C	ss50394787	1.1
*TGFBR2*: c.445-111A>G	rs17026161	2.2
*TGFBR2*: c.445-4T>A	rs11466512	21.7
^*a*^*TGFBR2*: c.1149G>A	ss50394788	1.1
*TGFBR2*: c.1157C>T	rs2228048	2.2
*TGFBR2*: c.1515-91C>A	rs2276767	4.3
*TGFBR2*: c.*327-329delAT	rs4016180	21.7
*TGFBR2*: c.*747C>G	rs11466531	7.6
*TGFBR2*: c.*835C>A	rs17026332	2.2

^*a*^Novel SNPs accepted by dbSNP

The distribution of genotypes was found to be in Hardy-Weinberg equilibrium for all SNPs in both case and control groups. No significant differences were observed in genotype and allele frequencies between case and control groups for any of the SNPs assessed (Table 7). Logistic regression analysis for the clinical characteristics described in Table 1 did not reveal a significant association with any variant and diabetic nephropathy. Adjusted *p *values for these potential covariates are shown in Table 8.

**Table 7:** Genotype and allele frequencies of selected SNPs in case and control groups. The five *TGFB1 *SNPs were selected on the basis of previous publications [23-27]. The remaining two *TGFBR2 *SNPs were selected from potentially functional SNPs identified through screening the gene as summarised in Table 1. Data are n (%)

**SNP**	**Genotype**	**Case**	**Control**	***p *value**	**Allele**	**Case**	**Control**	***p *value**
***TGFB1 *-800G>A**	GG	188 (69.1)	268 (73.0)	0.56	G	454 (83.5)	628 (85.6)	0.30
	GA	78 (28.7)	92 (25.1)					
	AA	6 (2.2)	7 (1.9)		A	90 (16.5)	106 (14.4)	
***TGFB1 *-509C>T**	CC	179 (65.8)	245 (66.8)	0.62	C	442 (81.3)	595 (81.1)	0.93
	CT	84 (30.9)	105 (28.6)					
	TT	9 (3.3)	17 (4.6)		T	102 (18.7)	139 (18.9)	
***TGFB1 *+72InsC**	- C	221 (81.3)	301 (82.0)	0.88	- C	488 (89.7)	663 (90.3)	0.71
	+/- C	46 (16.9)	61 (16.6)					
	+ C	5 (1.8)	5 (1.4)		+C	56 (10.3)	71 (9.7)	
***TGFB1 *+869T>C**	TT	151 (55.5)	204 (55.6)	0.86	T	403 (74.1)	540 (73.6)	0.84
	TC	101 (37.1)	132 (36.0)					
	CC	20 (7.4)	31 (8.4)		C	141 (25.9)	194 (26.4)	
***TGFB1 *+915G>C**	GG	219 (80.5)	298 (81.2)	0.98	G	488 (89.7)	661 (90.1)	0.84
	GC	50 (18.4)	65 (17.7)					
	CC	3 (1.1)	4 (1.1)		C	56 (10.3)	73 (9.9)	
^***a***^*TGFBR2 *c.*747C>G	CC	218 (90.5)	287 (89.1)	0.88	C	457 (94.8)	606 (94.1)	0.61
	CG	21 (8.7)	32 (9.9)					
	GG	2 (0.8)	3 (0.9)		G	25 (5.2)	38 (5.9)	
^***a***^***TGFBR2 *c.1149G>A**	GG	232 (96.3)	317 (98.4)	0.10	G	473 (98.1)	639 (99.2)	0.10
	GA	9 (3.7)	5 (1.6)					
	AA	0	0		A	9 (1.9)	5 (0.8)	

^*a *^272 cases and 367 controls were genotyped for TGFB1 SNPs with 241 cases and 322 controls genotyped for TGFBR2 variants.

**Table 8:** Significance values following logistic regression analyses adjusting the association between diabetic nephropathy status and genotype for the listed potential confounders.

***p *value adjusted for:**	***TGFB1 *-800G>A**	***TGFB1 *-509C>T**	***TGFB1 *+72InsC**	***TGFB1 *+869T>C**	***TGFB1 *+915G>C**	*TGFBR2 *c.*747C>G	***TGFBR2 *c.1149G>A**
**-**	**0.56**	**0.62**	**0.89**	**0.86**	**0.98**	**0.88**	**0.11**
Gender	0.56	0.45	0.98	0.92	0.94	0.88	0.09
Age at Diagnosis	0.56	0.61	0.88	0.86	0.98	0.88	0.11
Duration of Diabetes	0.62	0.69	0.94	0.94	0.98	0.56	0.26
HbA1c	0.44	0.63	0.96	0.80	0.97	0.86	0.16
BMI	0.79	0.72	0.63	0.79	0.97	0.22	0.72
Mean BP	0.94	0.93	0.68	0.99	0.77	0.31	0.85
All of the above	0.36	0.94	0.93	0.70	0.96	0.49	0.95

**Figure 1:**
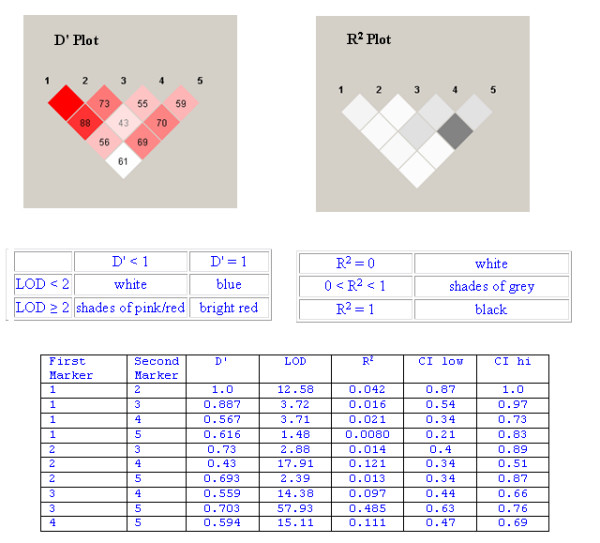
Although |D'| values were not particularly large for TGFB1 markers (D' Plot), they were statistically significant. R^2 ^measure, there was little correlation observed between the genotyped markers (R^2 ^Plot). Further details are displayed in the descriptive shown tables below the LD Plots. D' is the value of D primer between the two loci; LOD is the log of the likelihood odds ration (a measure of confidence in the value of D'); R^2 ^is the correlation coefficient between the two loci and CI low/CI high represent 95% confidence limits for D' where the minor allele frequency is greater than 5%.

**Figure 2:**
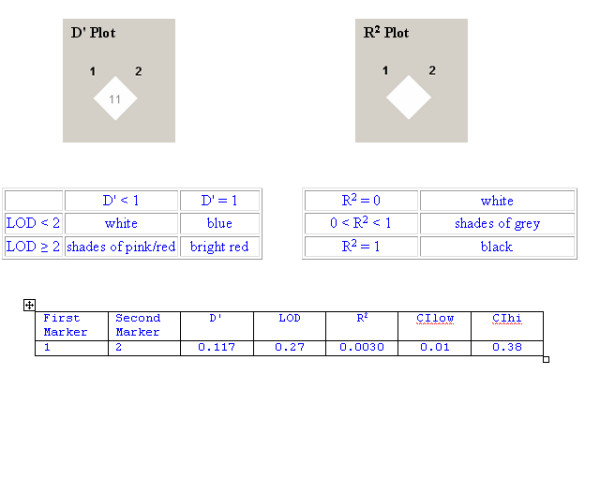
Strong linkage disequilibrium was not observed between the two genotyped markers for *TGFBR2*. Further details are displayed in the descriptive tables shown below the LD Plots.

The level of observed LD between all genotyped variants within each gene, together with the raw |D'| and R^2 ^scores are shown in the Figures (Figures 1, 2). The most common combinations of alleles observed 5' to 3' were GC-TG (49.4%); AC-TG (12.6%) and GT-CG (10.5%). In addition, we have identified a novel, rare variant at *TGFB1: *+728T>C (ss50394786).

## Discussion

There is considerable evidence supporting the role of *TGFB1, TGFBR1 *and *TGFBR2 *genes in the development of diabetic nephropathy. The functional importance of the TGFβ1 protein is illustrated by the high degree of conservation observed across mammalian species. The TGFβ1 gene comprises seven exons separated by relatively large introns [23]. Transcriptional regulation is complex with two major start sites identified at positions +1 and +271 [24]; polymorphic positions are typically reported relative to the first major transcription start site for this gene. We have assessed five SNPs, suggested to play a role in TGFβ1 expression levels through their impact on regulatory regions of *TGFB1 *[25-27], for association with diabetic nephropathy. Genotype and allele frequencies were similar to those reported in other European populations, with a decreased frequency observed for the minor alleles of *TGFB1: *-509C>T and *TGFB1: *+869T>C compared to the ECTIM results [25,28,29]. As illustrated by the LD Plots (Figures 1, 2), we observed low correlation between the genotyped SNPs. The |D'| values suggest that these SNPs have been separated by recombination, however as the magnitude of |D'| is biased for smaller sample sizes |D'|, lower values may be difficult to interpret. To further clarify the patterns of LD between genetic markers, we also examined R^2 ^values as this measure of LD is not as susceptible to small sample sizes. It is important to note that |D'| is less than one only when all haplotypes are observed and that markers with disparate alleles frequencies will also affect these measures of LD. The patterns of LD indicate that all genotyped loci within each gene are not contained within a single haplotype block, thereby suggesting limited value in performing haplotype analysis – i.e. the LD values suggest that these markers are not transmitted on a single haplotype, but are separated by recombination events. Our analysis suggests that all SNPs genotyped in this study are informative and the most common combinations of alleles observed are similar to those previously published [29]. Comparison of our data with Phase II, release 21 of the HapMap resource [30] supports our findings that the *TGFB1 *gene is located in a genomic region affected by a recombination hotspot. All recorded polymorphic sites in the HapMap CEPH (Utah residents with ancestry from northern and western Europe) population demonstrate poor correlation, suggesting that all should therefore be genotyped as 'tag' SNPs. None of the common variants genotyped for the *TGFB1 *gene in this study are recorded as polymorphic in the Phase II, release 21 HapMap Caucasian data set and the nearest polymorphic SNP is approximately 2 kb from our SNPs of interest.

We observed no significant differences in genotype or allele frequencies between case and control groups for any of the SNPs assessed. The *TGFB1: *+869T>C SNP has been associated with diabetic nephropathy in a Chinese population with type 2 diabetes [31], however our results do not support this finding for nephropathy in type 1 diabetes. The results from Wong and colleagues' smaller Chinese study (cases, n = 58; controls, n = 65), may be explained by a difference in genetic factors between type 1 and type 2 diabetes or differences between the Chinese and Irish populations.

Our study employed rigorous phenotypic criteria for inclusion of cases and controls. The annual incidence of diabetic nephropathy increases over the first fifteen to twenty years duration of type 1 diabetes, but after twenty-five years the absence of overt proteinuria makes the subsequent development of nephropathy unlikely [32,33]. The present report utilised cases and controls that were well matched for prolonged duration of diabetes (cases mean duration = 26.9 ± SD 8.3 years, control mean duration = 27.7 ± SD 9.0years). Our results for *TGFB1 *are in accord with Ng and colleagues' study which also failed to find an association between the *TGFB1: *-800G>A, -509C>T, +869T>C or +915G>C polymorphisms and diabetic nephropathy in US Caucasians with type 1 diabetes [34]. This is in contrast to a larger study in UK Caucasians where a significant association (p = 0.027) was identified between *TGFB1: *+869T>C and diabetic nephropathy [35]. This UK study utilised the Golden Years cohort of type 1 diabetic individuals as a control population [31] with all the recruited subjects (n = 410) having a very long duration of type 1 diabetes (> 50 years). Although these individuals did not have renal failure due to diabetic nephropathy 29% were taking antihypertensive medication and 35.7% had evidence of micro- or macroalbuminuria [36]. These clinical features (antihypertensive medication and micro- or macroalbuminura) form distinct exclusion criteria from our own diabetic control group. Although our sample size has ~90% power to detect a doubling in the minor allele frequency in cases relative to controls (*e.g*. 10% vs. 5%), there is a need for a collaborative genotyping effort in larger sample collections to definitively determine the role of these SNPs in predisposition to diabetic nephropathy.

TGFβ type I receptors form a heterodimeric complex with TGFβ type II receptors and bind to TGFβ to mediate many TGFβ activities including regulation of cell proliferation, differentiation and extracellular matrix production. It has been recently reported that TGFβ1-mediated epithelial-to-mesenchymal transition requires functional *TGFBR2 *[37]. Variants have been recorded for both *TGFBR1 *and *TGFBR2 *genes, however there is limited genomic information regarding their influence on diabetic nephropathy. *TGFBR1 *is composed of nine exons and maps to chromosome 9q33-q34 [38]. TGFBR2 is composed of seven exons and maps to 3p22 [39]. There are presently 409 validated SNPs recorded in dbSNP for these two genes (*TGFBR1*, n = 115; *TGFBR2*, n = 294; dbSNP, accessed 12/01/06). Due to the large number of reported SNPs and potential ethnic variation in SNP occurrence and frequency [40], we resequenced these genes in our population.

We prioritised screening of the protein coding regions of these genes to aid identification of potentially functional gene variants. We screened for variants directly affecting lariat regions, splice sites, exonic/intronic splice enhancers, signal sequences, protein coding sequence, polyadenylation signals and untranslated regions. It is possible that other variants may affect regulatory mechanisms, (promoter or enhancer elements, microRNA *etc*.) or that features such as post-translation modifications may affect these candidate genes and their subsequent protein activity. It is also possible that rare variants may play a role in susceptibility to diabetic nephropathy; however this study lacks sufficient sample numbers to definitively assess the role of rare variants in this disease. Five novel variants were identified in *TGFBR1*, of which none were at sufficient frequency to assess in this case-control collection. We have identified nine SNPs in *TGFBR2*. Two SNPs are located in exons (*TGFBR2: *c.1157C>T, *TGFBR2: *c.1149G>A) and one SNP in the 3' UTR which was found to be putatively functional (*TGFBR2*: c.*747C>G). Analysis of *TGFBR2*: c.*747C>G genotyping did not reveal a significant association with diabetic nephropathy. A microsatellite [AT]_del _was also identified in the 3' UTR of *TGFBR2*. *TGFBR2: *c.1157C>T in exon four was found in only one sample in the heterozygous state (MAF: 1.1%), and does not lead to a change in amino acid (aac → aat = N^389^N). *TGFBR2: *c.1149G>A was found in only two samples (MAF: 2.2%), but leads to a non-synonymous change in amino acid (gtg → atg = V^387^M) in the serine-threonine protein kinase active domain of the mature chain for TGFβR2 (PROSITE: PS00108; Pfam: PF00069, accessed 03/02/06). Genotyping *TGFBR2: *c.1149G>A did not reveal a significant association with diabetic nephropathy, however we did identify a doubling of the minor allele in cases (MAF: 1.9% in cases vs. 0.8% in controls). This finding may be due to the low frequency of minor allele, however our available sample numbers do not provide sufficient power to appropriately assess the association of this SNP with diabetic nephropathy. The search for causative variants for susceptibility to diabetic nephropathy is constrained by limited numbers of well-characterised, precisely phenotyped cases and controls, which represents a major challenge in the study of complex disease genetics. While the power to identify disease gene loci is influenced by many factors, the requirement for adequate samples sizes of stringently phenotyped individuals is critical to the success and validity of complex disease association studies. Our results warrant further investigations of rare variants, particularly the *TGFBR2 *exonic SNPs, provided the sample population is sufficiently powered to assess the association.

## Conclusion

Although experimental evidence suggests TGFβ1 blockade may be an important therapeutic target we were unable to identify any association between *TGFB1 *gene variants and diabetic nephropathy. In resequencing the genes we identified eight novel variants for *TGFB1, TGFBR1 *and *TGFBR2 *genes but did not detect significant association between any of the common SNPs and nephropathy in this Caucasian population with type 1 diabetes.

## Abbreviations

*MAF*: Minor allele frequency, *SNP*: Single nucleotide polymorphism, *UTR*: Untranslated region, *TGF*: Transforming growth factor

## Competing interests

The author(s) declare that they have no competing interests.

## Authors' contributions

AJM: Contributed to the conception and design of this study, carried out all resequencing and genotyping work, submitted all data to online repositories, drafted the manuscript and approved final manuscript.

DAS: Participated in study conception and design, critically reviewed the manuscript and approved final manuscript.

CCP: Performed all statistical analyses, contributed to data interpretation, critically reviewed the manuscript and approved final manuscript.

DS: Provided clinical supervision of patient recruitment in the Republic of Ireland, critically reviewed the manuscript and approved final manuscript.

APM: Participated in study conception and design, supervised recruitment of patients in Northern Ireland, contributed to data interpretation, co-wrote the manuscript and approved final manuscript.

## Pre-publication history

The pre-publication history for this paper can be accessed here:


